# Stigma in Practice: Barriers to Health for Fat Women

**DOI:** 10.3389/fpsyg.2016.02063

**Published:** 2016-12-30

**Authors:** Jennifer A. Lee, Cat J. Pausé

**Affiliations:** ^1^College of Arts, Victoria UniversityMelbourne, VIC, Australia; ^2^College of Humanities and Social Sciences, Institute of Education, Massey UniversityPalmerston North, New Zealand

**Keywords:** fat stigma, obesity stigma, healthism, HAES®, health at every size, eating disorders, co-autoethnography, fat studies

## Abstract

In this paper, we explore barriers to health for fat people. By shifting the focus from what fat people do or do not do, neoliberal principles are replaced by a focus instead on structural and institutional policies, attitudes, and practices. This includes the impact of stigma on the health treatment and health-seeking behavior of fat people. For example, we consider the role that provider anti-fat attitudes and confirmation bias play in the failure to provide evidenced-based healthcare to fat patients. This is an autoethnographic paper, which provides the opportunity to read research from the perspective of fat scholars, framed by questions such as: can fat people have health? Is health itself a state of being, a set of behaviors, a commodity, a performance; perhaps the new social contract? As a co-written autoethnographic paper, one aspect of the evidence provided is the recorded experiences of the two fat authors. This includes writing from notes, journals, compiled and repeated experiences with medical professionals, family, and the community. Framed by feminist standpoint and supported by literature drawn from Fat Studies, Public Health, Obesity Research, and other interdisciplinary fields, this is a valuable opportunity to present an extended account of fat discrimination and the impact of the stigma fat people face through the medical profession and other sectors of the community, written by fat individuals. The paper concludes by considering the health pathways available to fat people. Special attention is paid to whether Bacon and Aphramor's Health at Every Size paradigm provides a path to health for fat individuals.

Most people want to be healthy, but for many, there are barriers. These barriers can include structural oppression, historical factors, cultural approaches, economic conditions, individual needs, and more. In this paper, we attempt to answer the question, “What are the barriers to health for fat people?” We draw from theory, literature, social media, and our own experiences to provide an answer. We initially discuss health equity and disparity that leads to fat stigma, then we explore three barriers to health for fat individuals. The first barrier to health we consider is how health is defined and prioritized, which includes a discussion on who is allowed to be healthy and “healthism.” This leads into a critique of health provider bias and then the resulting patient avoidance of health care. These barriers are then applied using the case of eating disorders, as an example of how these barriers can converge and lead to a reduction in the mental and physical health of fat people. The paper finishes by considering whether Health at Every Size® (*HAES*®) provides a path to health for fat people.

To achieve this, we have used collaborative autoethnography (Geist-Martin, 2010) to allow us to consider what barriers we perceive, as fat women and as scholars, to health for fat people. Autoethnography is especially appropriate for this scholarship, as it allows for the researcher to remember and reflect on personal experiences, and engage in analysis of said remembrances through the lens of theory and literature (Ellis et al., 2011). As noted by Ngunjiri et al. (2010), autoethography “utilizes data about self and its context to gain an understanding of the connectivity between self and others within the same context” (p. 1). For this collaborative autoethnography, we each wrote narratives addressing barriers to health, definitions of health, and HAES®, among others. We shared these, and spoke about the apparent similarities and differences. Ngunjiri et al. (2010) suggested that this process contributes to the richness of the method; “their interactions produced a richer perspective than that emanating from a solo researcher autoethnography. One researcher's story stirred another researcher's memory; one's probing question unsettled another's assumptions; one's action demanded another's reaction” (p. 6). We sought the answer to our research question as we continued to write and develop our perspectives, while engaging with the literature, both scholarly and lay, along the way.

We are feminist scholars, working in different areas of scholarship; Jenny is from creative arts and Cat is from social science. The integration of our two voices and scholarship styles makes this manuscript unique to others in this special issue. Another aspect that sets this paper apart is that we are both fat women who embrace fat politics in our lives, both personally and professionally. We identify as fat activists, and our belief that fat people deserve the same rights and dignity as non-fat people will be seen throughout this work. Our use of the word fat, for example, sets us apart from scholars who accept the word “obesity” and the pathologisation of body size. And our acknowledgment that fat people can be knowers about fatness locates us in an epistemological standpoint quite different from most who conduct obesity research.

Autoethnographers do not claim to be entirely objective. Autoethnography allows authors to critically reflect on experience, privilege, and to admit the subjectivity of their position (Ellis et al., 2011). This is a different position to obesity researchers and their monolithic methodology, where subjectivities supposedly do not exist, and theories about obesity are “proven” rather than “tested.” As Charlotte Cooper highlights,

Obesity discourse is totalitarian, by which I mean it presents itself as the only authority on fat, nothing else counts. Fat is a crisis brought about by a mismanagement of energy balance, it offers nothing of value, it is only an opportunity for intervention. It is always about health, and health is presented as an apolitical fact (Cooper, 2016, p. 24).

The relationship we have to the text of this paper is different to a paper that is presented as an objective analysis of findings. Therefore, the reader's expectations of this paper need to be different. We reveal our relationship to the material and go beyond that to use stories from our lives to illustrate points made about fat and stigma and barriers to health.

The topic of this special issue is “Obesity stigma in healthcare: impacts on policy, practice, and patients.” This is a timely special issue, as governments across the world are setting goals to reduce obesity rates at the same time as they set other goals to address health inequities. Equity in health, according to Braveman and Gruskin (2003) is the “absence of systematic disparities in health (or in the major social determinants of health) between groups with different levels of underlying social advantage/disadvantage” (p. 254). In practical terms, equity in health means “equal opportunity to be healthy, for all population groups/resources are distributed and processes are designed in ways most likely to move toward equalizing the health outcomes of disadvantaged social groups with the outcomes of their more advantaged counterparts” (p. 257). Whitehead (1992) suggests that “equity in health implies that ideally everyone should have a fair opportunity to attain their full health potential, and more pragmatically, that none should be disadvantaged from achieving this potential” (p. 433). These groups are most often delineated along lines of class, ethnicity, gender, and geography. We argue that these “have” and “have nots” in regards to health can also be understood as those who are fat and those who are not. The systematic disparities in health experienced by fat people meet the criteria suggested by Starfield (2001); the associations are persistent and significant.

## Health disparities and fat stigma

Fat individuals are less likely to access healthcare, and are less likely to receive evidence-based and bias-free healthcare when they do engage (Drury et al., 2002; Aldrich and Hackley, 2010; Forhan and Salas, 2013; Phelan et al., 2015). For example, fat ciswomen are less likely to receive cervical cancer screening (Adams et al., 1993; Simoes et al., 1999; Amonkar and Madhavan, 2002; Carney et al., 2002; Cohen et al., 2007), breast cancer screening (Wee et al., 2000; Østbye et al., 2005; Zhu et al., 2006; Mitchell et al., 2008), and colorectal cancer screening (Ferrante et al., 2006) than non-fat ciswomen; this is especially true for super fat ciswomen (Ferrante et al., 2010). Fat ciswomen with breast and cervical cancers are more likely to die from breast and cervical cancer than non-fat ciswomen with these cancers (Hunt and Sickles, 2000; Wee et al., 2004; Aldrich and Hackley, 2010).

Cancer screenings are not the only cases where fat individuals have poorer access to healthcare. Similar inverse relationships between BMI and preventative services have been documented in relation to the influenza vaccine in the elderly (Østbye et al., 2005), for example. In Canada, Lavoie et al. (2006) found a positive correlation between BMI and asthma control and quality of life, while controlling for asthma severity, sex, and age. The authors hypothesize that several behavioral factors may contribute to this, including the delay and avoidance of healthcare by fat individuals.

The health disparities experienced by fat individuals are clear; the question then arises as to whether they are unfair. Some might suggest that we would expect fat individuals to experience poorer health than non-fat individuals. This would certainly be supported by most of the empirical research produced by obesity researchers. What if, however, we removed the “everyone knows being fat is unhealthy” assumption and explored whether these disparities are instead a result of the systematic and structural oppression experienced by fat people? What if, for example, the impairment to health for fat people is located within the social stigmatization of fat people? We aren't the first to ask these questions (i.e., Muenning, 2008), but we do believe we have much to add to the conversation.

Fat stigma is placed upon fat people due to the negative associations and stereotypes associated with fatness (Pausé, 2012, 2014). Goffman (1963) argued that stigma is attached to aspects of an individual that are “deeply discrediting,” including “tribal stigmata,” “blemishes of individual character” and “abominations of the body” (p. 3). Fat people can be said to be stigmatized along the latter two; fatness is seen as a blemish of character. And fat bodies are marked as abominations (DeJong, 1980).

Stigmatizing attitudes of fatness^1^ are found across the world. Brewis et al. (2011) collected data from 600 adults across 10 countries, including Iceland, New Zealand, Tanzania, and American Samoa, and found anti-fat attitudes are pervasive and have infiltrated cultures that once did not demonstrate anti-fat attitudes. Stigmatizing attitudes are found in children (Latner and Stunkard, 2003; Wills et al., 2006; Eriksen and Manke, 2011; Barlösius and Philipps, 2015), adolescents (Wills et al., 2006; Barlösius and Philipps, 2015) young adults (Ambwani et al., 2014), and adults (Greener et al., 2010); over one-third of young adults in Ambwani et al. (2014)'s research agreed that, “one of the worst things that could happen to a person would be for [them] to become obese” (p. 368).

Stigmatizing attitudes are often multidimensional. For example, Ambwani et al. (2014) found that stigmatizing attitudes held both that fat people suffered from their fatness, and that fat people were inferior to non-fat people because of their fatness. Stigmatizing attitudes are held by fat people as well; most internalize the anti-fat attitudes of the culture in which they live (Rogge et al., 2004; Greener et al., 2010; Lewis et al., 2011; Hilbert et al., 2014). Fat stigma is (re)produced through the family (Puhl and Brownell, 2006; Puhl and Latner, 2007; Ogden and Clementi, 2010), the media (Himes and Thompson, 2007; Heuer et al., 2011; Brown and McClimens, 2012), and healthcare providers (Puhl and Brownell, 2006). It is also (re)produced through relationships and interactions with friends (Puhl and Brownell, 2006; Puhl and Latner, 2007), co-workers (Puhl and Brownell, 2006; Sadati et al., 2016), classmates (Puhl and Brownell, 2006; Sadati et al., 2016), and teachers (Puhl and Latner, 2007). For many fat people, fat stigma is a daily part of their life.

While most research on fat stigma has been conducted in the United States and the United Kingdom, scholars have found anti-fat attitudes in Germany (Stein et al., 2014), Dominica (Council and Placek, 2014), and Turkey (Dedeli et al., 2014), among others. As noted earlier, Brewis et al. (2011) found anti-fat attitudes across young adults in 10 countries, with the highest anti-fat attitudes in Paraguay, American Samoa, and Mexico (as opposed to the United States or United Kingdom, as many might assume). In their work in Dominica, Council and Placek (2014) note that anti-fat attitudes were highest among individuals who were involved in social media networking sites like Facebook, demonstrating the colonizing effects of the World Wide Web (Troumbley, 2013).

Fat stigma is a powerful driver of population level health disparities (Muenning, 2008; Stuber et al., 2008; Hatzenbuehler et al., 2013; Hunger et al., 2015). Puhl and Heuer (2010) argue, “[fat] stigma is not a beneficial public health tool for reducing obesity or improving health. Rather, stigmatization of obese individuals pose serious risks to their psychological and physical health, generates health disparities, and interferes with implementation of effective obesity prevention efforts” (p. 1019). While fat stigma results in many barriers to health for fat people, as stated earlier, we have chosen to focus on three: how we define and prioritize health, the anti-fat attitudes of healthcare providers, and the resulting avoidance of healthcare by fat individuals.

## How health is defined and prioritized

The first barrier to health for fat people is how health is defined and prioritized. The World Health Organisation defined health in 1948 as “a state of complete physically, mental, and social well-being and not merely the absence of disease or infirmity.” This definition moves health away from a state of being free of disease, to a label awarded to those who are able to negotiate their way through multiple physiological, behavioral, and social criteria. First applauded for recognizing that health was more than being free from disease, the WHO definition is now criticized for moving all aspects of life into the health realm (Callahan, 1973), supporting the medical industrial complex. Huber et al. (2011) argue that the WHO's definition of health is no longer “fit for purpose” as populations live longer, and develop an increasing number of chronic illnesses in the process (p. 1). The Ottawa Charter suggested that health was best understood as a resource, not an objective (World Health Organisation (WHO), 1986).

As Metzl and Kirkland (2010) query, in any definition of health, who is left out? Jenny reflects on the notion of who can be healthy:

*Because I have Type 2 diabetes, I imagine that doctors would not consider me a good example of a fat person who is healthy. Although this notion of “healthy” is a strange one anyway, as if the body and mind are a machine that can function perfectly if we try hard enough. What permanently puts you in the category of “unhealthy,” beyond being fat or having a fat-related illness? A slim friend of mine who has a spinal injury and has had several surgeries and has to ration her pain medication? Is she considered “healthy” or “unhealthy”? Like “normal,” “healthy” brings with it certain assumptions, and idealising a healthy state is problematic*.

To declare health a state of complete physical, mental, and social well-being, leaves a great deal of people out from having health. Anyone with a chronic illness or disability, for example, is left out of that definition; our friend in a wheelchair; our colleague who is HIV positive. Anyone living with a mental illness, or without a support network of family and friends, are left out of that definition; our aunt with anxiety; our neighbor who doesn't have a support system. And fat people, presumably, are left out of that definition. Is a person's health understood as their BMI? Perhaps having a “healthy” BMI is simply one threshold of many to having health. But if that is the case, then why would fat people give much thought to their health in any holistic and meangingful way, if it is a state they are denied because of their body size? Cat has been asking these kinds of questions about the public and private perceptions of health for fat people, and for herself:

Is health important to me? How do I understand what health means as a fat person? I think I want to be in good health. I want to live a fully functioning life, and I understand good health to be part of this equation. But I often get discouraged, because others will not allow me to have health. Ever. Because I am fat. And part of me wonders—what is even the point? So how do I understand what health means for me? Or if I should even bother?

*My view on health has shifted quite drastically over the years. The last time I went to war with my body, my motivation was vanity. I was in my first significant romantic relationship, and I couldn't help but notice that slimmer girls would often catch his eye. So I decided I wanted to become smaller—become the non-broken girl I believed was inside myself. I decided to eat fewer calories and begin exercising, and I was rigid. I had lots of routines and lots of plans and lots of self-flagellation. Unsurprisingly, I lost weight. A lot of weight. And then I plateaued. As you do. And I panicked. And cut back even more calories. And began to exercise even more. I was exercising for up to four hours every day (I wonder about how in the world I completed my PhD during this time!) ….At this point, I decided to stop smoking. In my belief, I had taken my health under my control with my diet and exercise, and thus it was the right time to quit smoking and pursue another aspect of healthy living*.

*As my mental health deteriorated over the two years, I came to recognise that 198 lbs was as small as I was gonna get under my own volition. 198 lbs. This was still 98 lbs more than I was “supposed” to be. Was I healthy? I was fit—I was eating an incredibly considered diet. I was no longer a smoker. But I wasn't healthy—my BMI still put me squarely as obese. And I was miserable. Completely miserable. My partner and I broke up; so I was alone and miserable. But healthy? Maybe. Thinner? Definitely. I think we broke up because I was unhappy with myself and my body (which I wasn't when we got together), and that made it hard for anyone else to be happy with me and my body. I tried to shift my thinking away from the scale and onto my health, but it was an impossible task. I didn't know any other way to assess whether I was making progress—whether I was bettering myself with all my sacrifice—without using the numbers on the scales. My metabolic health wasn't useful, because that had always been “good.” I couldn't use how I felt about my body, or how my body moved through the day, because while I could see that my fitness had improved—my body moved through my days great before the war began. And I felt worse about my body now at 198 lbs than I had at 270 lbs*.

Part of me thinks that if I'm gonna implode by 50, regardless of anything else, then why bother pushing myself to engage in health seeking behaviours that don't add anything positive to my life? Part of me knows that no matter how “healthy” I am, it won't matter to anyone but maybe me and my loved ones (and even they might always want me to be “healthier”). Healthcare providers won't see past my body, strangers won't see past my body, colleagues won't see past my body. If I can never be healthy, then why do I buy into their idea in the first place?

Regardless of which definition of health is embraced, it must be recognized that ultimately, health is defined socially, by individuals, by cultures, by institutions (Burr, 2015). Understandings of health, and of good health, are shaped by the environments in which we operate. But there are many that would argue for a core aspect of health that is independent of sociohistorical time and place. Is there something essential to health? Something at the core that must be present for health—or something that must be absent?

When Cat was applying for residency in New Zealand, she noted that the application required a demonstration of an “acceptable standard of health” (New Zealand Immigration, 2015). This was further defined in the document by meeting three criteria: unlikely to be a danger to public health; unlikely to impose significant costs or demands on New Zealand's health services or special education services; and ability to perform the functions for which you have been granted entry.

Unlike countries such as the United States and Australia, New Zealand immigration did not clearly prohibit those over a set BMI threshold from applying to enter the country. In this way, Cat was required to complete the standard medical certificate as part of her application. While seeking residency several years after entry, she was required to complete another round of medical checks. And another. As she “passed” each check, new (lower) thresholds for acceptable health were set. After three rounds of completing, and passing these checks, she was deemed to not meet the acceptable standard of health, based on solely on her BMI.

*It was so incredibly frustrating; to undergo additional testing because they couldn't believe that someone so fat was metabolically healthy. A few weeks later, I received the decision from Immigration that I was denied residency because I did not meet the medical standard required. Based solely on my BMI. All of my medical tests had been good. Nothing raised any flags, or fell outside of normal parameters. But my BMI was over 30, so nothing else apparently mattered. And the belief that because of my BMI, I would end up costing up to $25,000 in medical care costs during my time in New Zealand*.

*Being denied residency based on my BMI was one of the most embarrassing things that had ever happened in my life. I mentioned this to a friend while the ordeal was dragging on, and she suggested that there was nothing to be embarrassed about; this was them showing their ignorance. While I appreciated her support and reframing of the event, it did nothing to lessen my mortification that I had been deemed too fat to stay in New Zealand (Cat)*.

Our understanding of health is sustained through social mores and processes (Burr, 2015). One of the best examples of social mores driving understandings of health is Cartwright's (1851/1981) diagnosis of drapetomania, the disease that caused slaves to run away from their owners. As technology advances and cultural perspectives shift, often it may seem that the tail is wagging the dog as thresholds for interventions are lowered, and conditions are only recognized once a drug is developed to address the condition (Sartorius, 2006; Huber et al., 2011).

Callahan (1973) suggests that part of the frustration with the WHO definition is that it requires recognition of the holistic nature of well-being, and “thwarts any movement toward a dualism of self and body” (p. 77). In this way, it could be suggested that attention paid on the health of fat people must go beyond concerns around body mass index and metabolic health. How is the mental health of fat people? How is their social well-being?

Given the anti-fat society that surrounds fat people, we can imagine that the mental and social health of fat people are at great risk; possibly greater risk than their physical health. And if we acknowledge that mental and social health have significant impacts on physical health? The entire approach to fighting the war on obesity would have to drastically revise and revisit its strategy. Health is often presented as something you “earn” or “maintain,” especially when it comes to weight. This is part of the notion that, if you eat well, “take care of yourself,” and exercise, you will be fit and healthy and thin, and live a long life. Unexpected illnesses are a “tragedy” because they're undeserved—by thin people. Even cancer is now possibly a fat person's fault—because they're supposedly more at risk of it. There is a moral judgment of deliberate choices to not prioritize physical health above everything—again, mainly if you're fat.

Cheek (2008) argued that health is “a new form of a badge of honor by which we can claim to be responsible and worthy both as citizens and individuals” (p. 974). In an increasingly neoliberal West, individual responsibility has become the hallmark of government policy, philosophical frameworks, and moral underpinnings. This individual responsibility is characterized by those who manage and maintain personal well-being and success; be it in employment, housing, education, personal relationships, and health. Those who require support, especially those who cannot proffer an acceptable reason, are irresponsible; failed citizens. Health has become the new social contract (Pausé, 2015). Neoliberal cultures promote individual responsibility for maintaining individual health; individuals have a moral obligation to one another to be healthy. “Health is a term replete with value judgments, hierarchies, and blind assumptions that we speak as much about power and privilege as they do about well-being “…”appealing to health allows for a set of moral assumptions that are allowed to fly stealthily under the radar” (Metzl and Kirkland, 2010, p. 1–2).

Healthism, as a term, was coined by Crawford (1980) to “represents a particular way of viewing the health problem, and is characteristic of the new health consciousness and movements” (p. 365). Cheek (2008) posited that healthism is an offshoot of conservative ideology; rooted in the belief that people reap what they sow. For some, healthism is a religion. We'd argue that healthism is also a form of oppression based on actual or perceived health status. As noted by Callahan (1973), “what can no longer be done in the name of “morality” can now be done in the name of ‘health”’ (p. 84).

How we understand health, and who is allowed to have health, is central to the health and well-being of fat individuals, especially as health (or their perceived lack of health) is used as a weapon against fat people in their fight for civil rights. On the front lines of health, and the “war on obesity,” are healthcare providers. Anti-fat attitudes in healthcare providers are incredibly well-documented (Hebl and Xu, 2001; Puhl and Brownell, 2001; Budd et al., 2011; Johnston, 2012; Forhan and Salas, 2013).

## Anti-fat attitudes of healthcare providers

Anti-fat attitudes are found in both practitioners and students, from physicians/doctors (Sabin et al., 2012), researchers (Schwartz et al., 2003), clinicians (Schwartz et al., 2003), physiotherapists (Setchell et al., 2014), dieticians (Stone and Werner, 2012), and nurses (Poon and Tarrant, 2009); to students in the medical and dietectic fields (Berryman et al., 2006; Poon and Tarrant, 2009; Persky and Eccleston, 2011; Phelan et al., 2014). These anti-fat attitudes are most likely products of the anti-fat attitudes in the larger society, and are especially influenced by neoliberal ideologies around individual responsibility. Healthcare providers perceive fat individuals as failed citizens (Jeffrey and Kitto, 2006; Stone and Werner, 2012) and less likely to be compliant with healthcare instructions (Hebl and Xu, 2001; Mercer and Tessier, 2001; Wigton and McGaghie, 2001; Foster et al., 2003; Brown et al., 2007; Persky and Eccleston, 2011). Ferrante et al. (2006) suggests that as healthcare providers are likely to believe that fat patients are less concerned about health, and less likely to be medically compliant with healthcare instructions, they may believe them to be less interested or worthy of preventative care. Cat experienced this dismissal by an endocrinologist she was forced to see during her immigration process,

*I was instructed [by Immigration] to complete an additional set of medical tests, and to see an endocrinologist. I remember my appointment with the endocrinologist very well. There is only one in the town where I live, so getting the booking took some work. On the night of my appointment, I arrived at his practice, and spent some time chatting with his wife about my time in New Zealand. I remember her dismissing my chances of residency based on my BMI, and her incredulous responses to my suggestions that being fat (even as fat as me) was not the worst thing, or the unhealthiest thing, in the world*.

*When I entered her husband's exam room, he had already completed most of his evaluation form for immigration. He had me change into a gown that was too small, and sit on the exam table while I waited for him to return. The paper crackled underneath my fat ass. He did not have a large BP cuff, so he was unable to take my blood pressure; he raised an eyebrow at my assertion that it was normally 110/70. He listened to my heart, checked my reflexes, and told me I could get dressed*.

*When I emerged fully clothed from the changing room, he was filling out the blanks in his template form. He didn't ask me anything about my health, or history, or health behaviours. He told me that while my blood work was all well within normal limits, that wouldn't last long and I would be diabetic before I was thirty. He dismissed me with a nod of his head, and I left the office*.

The anti-fat attitudes held by providers present yet another barrier for fat people in accessing appropriate healthcare. Negative attitudes about fat patients result in doctors choosing to spend less time with fat patients (Maroney and Golub, 1992; Foster et al., 2003; Hebl et al., 2003; Persky and Eccleston, 2011; Stone and Werner, 2012) and often resulting in fewer preventive and diagnostic tests for fat patients; doctors often report that these tests are more challenging with fat bodies (Adams et al., 1993; Amy et al., 2006; Ferrante et al., 2006, 2010).

Huizinga et al. (2009) found that fat patients were less respected by physicians than non-fat patients, and many doctors report that they prefer not to provide care for fat people (Maroney and Golub, 1992; Foster et al., 2003; Hebl et al., 2003; Persky and Eccleston, 2011; Stone and Werner, 2012). The primary care physicians in a study from Hebl and Xu (2001) reported an inverse relationship between patient BMI and the PCP's patience, job satisfaction, and willingness to assist the patient. Dieticians in Stone and Werner (2012) research expressed feelings of frustrations with their fat clients; “and this situation repeats itself, this frustration of people coming and not losing any weight. This frustration of knowing that I did my best, and I kept my end of the deal, and now it's your turn [the patient's]”(p. 771). Similarly, general practitioners in Australia reported working with fat patients as “professionally unrewarding,” due to the “limited efficacy in weight management” (Campbell et al., 2000, p. 459). Frank (1993) and Foster et al. (2003) found physicians to perceive treatment of obesity as futile.

In addition to personal bias against fat patients, healthcare providers may be less interested or able to tend to fat patients due to inadequate equipment (Zuzelo and Seminara, 2006; Ferrante et al., 2010; Destounis et al., 2011; Ingraham et al., 2014; Setchell et al., 2015) and their lack of education around caring for fat patients (Green et al., 2000; Amy et al., 2006; Brown et al., 2007). Whether due to improper equipment, lack of education, bias, or an interaction between the three, it is clear that providers also present a barrier to the health and well-being of fat individuals.

## Healthcare avoidance

This is further compounded by the resulting delays in accessing healthcare by fat individuals. For fat patients, the delay to access healthcare services may be explained by a range of factors that can often be attributed to healthcare avoidance (Bryne, 2008). These include avoidance of inadequate equipment such as gowns that are too small, exam tables/areas that are too small, and blood pressure cuffs that do not fit (Thompson and Thomas, 2000; Amy et al., 2006; Merrill and Grassley, 2008; Ingraham et al., 2014). Avoidance of healthcare settings may also be contributed to past advice around weight loss that was perceived as unhelpful to the individual (or unsolicited; Drury et al., 2002; Brown et al., 2006; Malterud and Ulriksen, 2010; Stephenson, 2011) and experiences of providers that held anti-fat attitudes (Merrill and Grassley, 2008; Stephenson, 2011; Buxton and Snethen, 2013; Forhan et al., 2013). Creel and Tillman (2011) reported that their participants experienced this bias through reluctantly provided care that was troubling to share even sometime later; “it was difficult for them to relate the hurtful comments, looks, and touches they had overheard or that had been spoken directly to them” (p. 1346).

Similarly, some may delay accessing healthcare because of a suspicion that regardless of their health needs, they will simply be diagnosed as fat and dismissed; other health concerns may not be heard or attended to (Amy et al., 2006; Brown et al., 2006; Merrill and Grassley, 2008; Creel and Tillman, 2011; Buxton and Snethen, 2013; Pausé, 2014; Lee, 2015); “that [the] physician would attribute all of their health concerns solely to their obesity” (Forhan et al., 2013, p. 369). Jenny reflects on the expectations of fat people when interacting with health professionals:

*When I interact with medical professionals, I have very little fear of physical pain—I don't have nightmares about the time I got coral stuck in my foot on a small island in Thailand and I didn't speak the language so the doctor didn't give me any pain killers for fear I was allergic (I think) and dug the coral out without pain relief. I dread seeing a new medical professional because I'm afraid they're going to judge me, talk about obesity, not treat me for my actual health condition, give me the wrong treatment because I'm fat, not listen to me, or flat out be mean to me. I told Cat about this and she said “we set the bar low.” She's right—the bar is so low, our expectations are so low, that pain is OK—hey, you want to stick a scalpel in me without pain killers—no worries—as long as you don't belittle me because I'm fat or keep telling me to lose weight. I'm grateful when all I get is physical pain, and I don't have to experience ridicule or misinformation or ignorance. The flip side of that is that I have put up with physical pain at times in my life in order to avoid the potential attitudes I might receive from a doctor. I've lived in my body for 36 years yet many medical professionals treat me as if I don't know anything about my own body or its needs (2013)*.

This is especially problematic for fat individuals who do have existing health issues that are considered co-morbidities with obesity. Jenny reflects on this here. For a full reflection on her diabetes diagnosis see “Not just a type: diabetes, fat, and fear” (Lee, 2012).

*Those of us who are fat are in a difficult position when we seek medical attention. We need expert advice, but we can't always be sure we'll get it. An example of that is when I saw a dietician who was supposedly an expert on diabetes and pregnancy and she focused entirely on weight loss, weighing me and announcing loudly “you have a BMI of 47.” She didn't give me any useful information about blood sugar, or about the importance of testing blood sugar when exercising. In fact, she discouraged my exercise efforts and said that “walking your dog isn't sufficient because he will stop and smell things or go to the toilet and then your heart rate will go down.” I had said I was trying to go to the gym twice a week and walk my dog on other days. Her final response was “you need to work out five times a week for an hour each time.” There was also no acknowledgment from her that walking my dog was good for me in many ways, as an academic who spends a lot of time on the computer or teaching, the fresh air, the sunshine, my dog's silly antics, the sense of “release” and “enjoyment” I got from it*.

*I told her I had an eating disorder background and had taken part in a moderate eating group and that I was aiming for healthy eating but not a weight loss focus. She ignored this and went on to show me how much rice I should be eating, using a cup she had in her office. I told her I cook, from scratch, a Thai curry about once a month. She asked, “Do you use full cream or low fat coconut milk.” I knew what the “correct” answer should be but I made a pact with myself not to lie to doctors any more—if you lie to them, aren't you possibly putting yourself and your health in danger? I told her I use full cream coconut milk and she told me I had to stop doing that. I stopped answering back and just left. But the real damage started after the appointment. For several months when I would eat something I knew she wouldn't approve of, I would think “fuck you, bitch”—clearly I felt disempowered, but this thinking was not pleasant for me, this anger was toxic. I think it is also telling that, for more than a year afterwards, I did not cook a Thai curry. I used to love breaking up the kaffir lime leaves from my garden, chopping coriander, juicing limes—the sensory pleasure of preparing a meal to share with my partner. She destroyed that pleasure for me for more than a year by implying that I was doing something wrong by eating something once a month that is OK for thin people to eat*.

*It took me a few months, but eventually I sent a complaint letter to the hospital about the dietician, and they wrote to me explaining that the dietician's supervisor had worked through my complaint with her. They offered me a different dietician but I declined, as I no longer trusted them. I found my own, Health at Every Size*® *trained dietician, through a website (Jenny)*.

Brown et al. (2006) found that some people may avoid healthcare settings because they have internalized fat stigma and believe themselves to be at fault for any poor health experienced, and unworthy of receiving treatment from providers. Far and away, the most common reason cited in the literature on the avoidance of healthcare by fat people is shame (Olson et al., 1994; Drury et al., 2002; Amy et al., 2006; Brown et al., 2006; Mitchell et al., 2008; Forhan et al., 2013; Pausé, 2014); it could be argued that many of the other reasons explored, including provider attitudes and being diagnosed as fat, are direct results of, or contributors to, the shame felt by many fat individuals. The shame experienced by fat people is a result of internalizing the anti-fat attitudes of the culture around them.

While we've considered these three barriers separately, we would suggest that most fat people experience these three barriers in an integrated fashion. How our providers perceive us as patients, as people, is largely shaped by the larger cultural views on health and weight and acceptability, and these cultural mores and individual provider attitudes are then embodied in the experiences fat people have when seeking treatment (from the too small chairs to the diagnosis of fat). One example in which this is best illustrated is through our cultural ignorance of fat people with eating disorders.

## Eating disorders: an illustration of the three barriers

In a review of what has been learnt about the causes of eating disorders, Culbert et al. (2015) found that eating disorders emerge through complex sociocultural, psychological and biological factors. They state in their findings that “Sociocultural idealization of thinness variables (media exposure, pressures for thinness, thin-ideal internalization, thinness expectancies)…attained “risk status” for eating disorders and/or disordered eating symptoms” (p. 1141). In addition, in cultures where overweight and obese people are stigmatized and women who are underweight are celebrated, many young women express dissatisfaction or disgust with their bodies. Polivy and Herman (2002) state, “This dissatisfaction often has emotional overtones of self-disgust. Body dissatisfaction, in fact, may be regarded as an essential precursor (and continuing accompaniment) of EDs. The more intense this dissatisfaction, the more likely that one will undertake attempts to lose weight” (p. 192). Therefore, while there are different aspects that contribute to the development of eating disorders, we will discuss how being fat, or the fear of becoming fat, in Western culture, contributes to eating disorders. Watkins and Hugmever (2012) write about how powerful it is for their students to consider the relationship between the “obesity epidemic” and the proliferation of EDs rather than viewing fatness and EDs as polar opposites (p. 173). In other words, if fat bodies were accepted and not hated in our culture, fat people would not embark on restrictive eating or disordered eating in order to lose weight, and the majority would not develop eating disorders. This fear of fat often develops in childhood and can be exacerbated by taunting, teasing or bullying related to being fat; a parent modeling dieting and internalized fat hatred; thin idealization, cultural and media influences, being weighed, and food being restricted (Presnell et al., 2004; McCabe and Ricciardelli, 2005; Culbert et al., 2015).

Assumptions and generalizations about fat people and their levels of health in Western culture are internalized by medical professionals. The results of recommending treatment for a patient based on an assumption rather than a clinical examination and discussion vary from: mis-diagnosis, no diagnosis, fat, or health shaming, a disbelieving attitude, an undermining attitude, and triggering an aversion in the patient to further seeking medical care—which was discussed earlier (Stafford et al., 1989; Kreuter et al., 1997; Pausé, 2014). Another aspect of failed medical treatment is when medical professionals respond to a fat patient as if weight is the only factor in that patient's health assessment. The doctor may fail to ask, or believe, the patient's description of eating and exercise habits (Jutel, 2005). In these cases, the doctor might assume the patient is sick because they are fat, or assume that any food restriction the fat patient does is positive, even if it involves the unhealthy thought processes and restricted eating-binge cycle of an eating disorder. The flipside of this is, a medical practitioner might treat otherwise “healthy” fat patients as if they are unhealthy, eat poorly or don't exercise because they are fat, and to blame any tangential conditions such as a sore knee or influenza on being fat. It also blinds medical practitioners when a fat patient does have a measurable medical condition such as high blood pressure or cholesterol, to blame it on their fat, with the simplistic cure being to lose weight at all costs (Bacon and Aphramor, 2011). With the notion of “lose weight at all costs” comes eating disorders. In addition, the “measurable medical conditions” are complex in themselves, as the points of measurement have shifted, as in the practice of diagnosing patients as “pre-diabetic”—with the cure supposedly being to lose weight (Jutel, 2014).

We argue that doctors are often unable to recognize when fat patients present with eating disorders. This isn't limited to healthcare providers; most of our culture fails to recognize that eating disorders, which are characterized through the cognitive processes relating to food, exercise, and the body, and the behaviors associated with eating disorders, can be enacted by a fat person (Whitelocks, 2013; McCarthy, 2014; Tait, 2015). It is possible to be “overweight” or “normal” weight and have anorexia—the condition is a set of thoughts and behaviors, usually resulting in weight loss—it is not a body type ((Burgard, 2004), p. 44). This is something that Cat discovered through a medical drama on television:

*The first time I ever realised that fat people could have eating disorders was while watching an episode of Grey's Anatomy. Everyone is scrambling to understand what happened to a patient, and Meredith (the ingénue intern) speaks up that the woman (the patient) is anorexic; her heart wall burst because her heart wall was weak due to her eating disorder. This hadn't occurred to anyone else, because the patient was a fat woman. But Meredith had been told by the woman's husband, that she had recently lost 100 pounds and was in the ‘best shape of her life’. The Fat Studies scholar and activist I am now would have been horrified to see an instance of weight bias play out in such a way, but at the time, knowing nothing about Fat Studies scholarship or fat politics, I was confounded that a fat woman, weighing 200 lbs, may have anorexia nervosa*.

It can be the case that fat people do not get their eating disorders diagnosed and do not receive referral to effective treatment. The stereotype that to have an eating disorder a person must be thin means that “overweight” or “obese” individuals are not diagnosed with eating disorders by some medical professionals. In England, one third of mental health trusts use BMI as the primary measure to decide whether to accept an individual as an eating disorder patient. BMIs in the “normal” weight range or above do not qualify (Donnelly, 2016; McCubbin, 2016). Bordo (2009) in “Not just a “white girl's thing”: the changing face of food and body image problems,” states:

Also left out of the “anorexic paradigm” were compulsive or binge eaters who do not purge, or whose repeated attempts to diet are unsuccessful. To have an “eating disorder,” according to the anorexic paradigm, means being thin—and since most compulsive eaters are overweight, it took a long time for clinicians to recognize that compulsive eaters, too, are suffering from an eating disorder (p. 80).

Another aspect of being a compulsive eater or binge eater that is rarely discussed is that the eating disorder can emerge from restricted dieting that had been attempted in order to lose weight. Carla Rice, in *How big girls become fat girls* discusses the results of her study on 21 ethno-racially diverse women, some with disabilities, aged 20–45 years old: “Whether they started secretive eating in childhood to resist restrictive diets or later adopted “disordered” eating to amend size differences disqualifying of their desirability, it is noteworthy that *all* participants perceived as fat eventually talked of taking up problem eating practices” ((Rice, 2009), p. 145). There can be cycles of restricted dieting, followed by binge eating, as the body's response to either physical hunger cues or psychological deprivation (Bacon, 2010). This restricted dieting usually begins as a response to body or fat hatred, and leads to weight loss, which is then followed by weight gain. Depending on the length of time each aspect of the cycle is lived, it can also result in weight cycling. Weight cycling is being acknowledged as damaging to health—potentially more damaging than remaining fat the whole time (Olson et al., 2000; Montani et al., 2006; Strohacker and McFarlin, 2010).

There is often an assumption that being fat describes particular actions and behaviors on the part of the fat individual. Kathleen LeBesco, in *Weight management, good health, and the will to normality* discusses this assumption. At times, being fat is “typically, reductively and mistakenly boiled down to the equivalent of “compulsive overeating,” a semantic collapse that does far more harm than good” (LeBesco, 2009, p. 191). LeBesco rightly critiques the notion that, while anorexia nervosa and bulimia are problems related to food, eating and the body, “obesity” is often considered a similar problem. However, “obesity” does not in fact describe a particular way of eating or living. The assumptions made about how “obese” individuals live their lives emerge from the ways in which health is defined in our culture. These limiting definitions of health, including the notion that to be healthy one must be in the “normal” BMI category, lead to notions that obesity is always caused by over-eating and under-exercising.

In addition to fat individuals not being diagnosed with eating disorders and not being referred to effective treatment, doctors may prescribe behaviors associated with eating disorders to their fat patients as the solution to their “obesity.” The cognitive processes and eating behavior that are observed in lower weight individuals that are judged to be eating disorders are often encouraged in fat patients (Burgard, 2004). Subsisting on extreme food restriction is often considered a positive behavior in fat patients—there the notion of how to measure health is often disregarded in favor of one single health measure—is the patient fat or not? Deb Burgard states:

If people have to do things in their day-to-day life to achieve a particular weight that a study says would be healthier—and the things that they have to do (like stomach surgery, starving, or exercising 4 h a day) are not compatible with loving self-care—then, by definition, that is not a “healthy” weight for that individual (Burgard, 2009, p. 43).

Burgard also argues, “Our own weight biases affect how we define eating disorders. One example is the low weight criterion for anorexia nervosa. Why should you have to wait to get diagnosed and treated for all the symptoms of AN till you are at a population-based, rather than an individually-based, “low” weight?” (Burgard, 2013). The cognitive and behavioral aspects of an eating disorder are often ignored in fat patients, in favor of encouraging weight loss (Burgard, 2006).

Jenny writes about the history of eating disorders in her family, and how fatness is viewed as the pathology:

*About 10 years ago I began three years of a mindful moderate eating program, which involved learning not to diet or binge, to eat mindfully, and to acknowledge all emotions. My binge eating disorder became well-managed over time. However, because I am fat, my parents believe they are the healthy ones and have implied in the past, that I am not. They have never had treatment for their disordered eating, dieting, bingeing or compensatory excessive exercise. My mum has won several prizes for maintaining her weight loss in her local low-cost weight loss group. But she often can't get out of bed in the morning from exhaustion, and still does two long walks a day, plus swimming on some days. My sister recently won a prize at her gym for losing the most fat and gaining muscle, however she says she is cold all the time, which is a symptom of being below your set point weight. Her weight has fluctuated over many years, going up and down. My dad skips meals so he can eat two desserts and rides his bike long distances. To be fair, my family no longer speaks to me about being fat, because I demanded they do not, though my mum looks me up and down sometimes. I have no idea if they are metabolically healthier than me. My mum suffers from unmanaged anxiety and OCD symptoms, which could manifest itself as physical health problems in future. Her GP just congratulates her on her weight loss, and keeping it off, without questioning how she does it, with the constant negative self-talk, low self-esteem, deprivation, banning of foods, and anxiety*.

In light of the disordered eating that can develop out of embodying fatness and interacting with Western culture and medical professionals, we have discovered that one of the largest barriers to the health of fat people is the notion of needing to “attain health” on the terms we are offered, within the dominant definitions of what it means to be healthy. The terms we are offered as fat individuals are narrow. The first set of terms has been discussed earlier in this paper; what we refer to as the health hierarchy in Western medicine. The health hierarchy in our culture prioritizes physical health above other aspects of health, such as social health and mental health. Health can be seen as the new religion, as the new social contract with rigid rules where people ask others about their health, aggressively wanting to know the health status of a fat individual, and chronic diseases correlated with being fat are demonized. Type 2 diabetes is the devil in this paradigm. With this health hierarchy comes health shame and fat shame, and the dogmatic notion that weight loss by “eating appropriately” and exercising is the most important aspect of health for a fat person. Cat writes about her attempt to exercise without a focus on weight loss:

*I remember a few years ago I decided I wanted to try my hand at circuit activities. There are several Curves gyms here in my town, and I was attracted to the idea of women-only spaces that encourage short bouts of activity across a range of circuits. I got so into this idea that I made an appointment at my closest location, and prepared myself for the usual “weight loss” BS—certain that I could engage with their programme without subscribing to the weight cycle industry that has ruined my relationship with diet and exercise across my fat existence. I thought I could approach it the same way I had finding a GP—by being clear at the fore that I wasn't interested in weight loss, but health, and by navigating my way through on my own terms. Imagine my dismay when I arrive for my appointment, and speak with the seller about my intentions—only to find that I cannot opt out of being weighed during each visit. I remember trying to explain to the individual why I didn't want to be weighed; how triggering I would find that; and being told that I couldn't participate in their programme without that aspect being included*.

Perhaps a better standard of care could emerge from a redefinition of what it means to “have health.” Currently in the Western world, certain types of people are labeled “unhealthy” and stigmatized. Instead, policy could support the opportunity for quality of life across the population, provide support to manage chronic illnesses, and treat disease in the best ways a society can. This also means accepting illness as a part of life and not assuming you can control your own, or others', health (Metzl and Kirkland, 2010).

## A path to health?

An alternative to the weight loss as health approach for fat people is the Heath at Every Size® approach (Bacon and Aphramor, 2011). HAES® shifts the focus off weight and onto health for all body sizes. HAES® rejects the use of weight, size and BMIs as a measure of health and instead advocates a holistic approach to health, including but also going beyond nutrition and movement. HAES® reconceptualises some commonly accepted notions of health, and instead positions health as being on a continuum and changing at different stages of an individual's life, of health as being a resource, and not a goal of living. HAES® principles are also against healthism: “Pursuing health is neither a moral imperative nor an individual obligation, and health status should never be used to judge, oppress, or determine the value of an individual” (ASDAH, 2016). The five principles of HAES® are weight inclusivity, health enhancement, respectful care, eating for well-being and life-enhancing movement (for a full review of HAES®, see Bacon, 2010). The HAES® approach has proven to be an effective method for producing improved physical health outcomes for fat people independent of weight loss (Bacon et al., 2005; Carroll et al., 2007; Provencher et al., 2007; Gagnon-Girouard et al., 2010; Tylka et al., 2014).

Jenny writes about her relationship with HAES®, 6 years after first discovering the HAES® principles:

*I find HAES*® *vital when interacting with the medical profession. It's a model I can take to a doctor, and it helps me to navigate how I want my treatment to look. Because of my frustration with how badly medical professionals have dealt with my body size in the past, I don't even want to discuss what I eat or what exercise I do, or even my weight in relation to having diabetes. I feel like wearing a sign to the doctors or to specialists that says “you only have permission to discuss the specific health issue I bring to you” and “you may not refer to my weight at all.”*

*Do thin people get up in the morning and think about the health principles by which they'll live their lives? Or do they assess, as I do, whether I can go to yoga when I have a work deadline and need to pick up my daughter from her Dad, as I have her that night? I'm sure other single parents who work a 55 hour week are with me there. My body feels better when I walk the dog, eat a salad, get a massage, and dance with my toddler. And I feel “stale” when I work a 14-hour day on the computer to meet a deadline. HAES*® *helped me make an agreement with myself to do movement I enjoy rather than constantly drop out of exercise programs I hated. It helped me to accept fluctuations in eating, and to do things that are good for me, and are about self-care. Perhaps I take HAES*® *for granted now, because I took what I needed from it. But it did help change my relationship to eating, movement, self-care, and perfectionism*.

HAES® illuminates and presents an argument about the ways in which health is defined and prioritized in Western culture. Unlike the WHO's definition that presents a notion of complete health that is the threshold individuals should aspire to “be healthy,” HAES® is a more inclusive model that does not delineate between who and who is not healthy. For example, tt contradicts common perceptions that fat people are not allowed to be considered “healthy.” HAES® has embedded, within its principles, the notion that health should not be defined by weight, or certainly not by weight alone (Bacon, 2010). It acknowledges that there are correlations between certain diseases and fat people, but questions where scientists and health professionals turn correlation into causation. It calls for a redefinition of health, to encompass physical and mental health, to focus on movement that is enjoyable and potentially relieves stress, and on work-life balance. HAES® principles suggest focusing on holistic healthy behaviors, throwing away assumptions about fat people not exercising or eating badly, and instead encourages all bodies to live as well as they can. HAES® advocates for striving for health, no matter what size you are (Bacon, 2010; ASDAH, 2016).

In that sense, if applied in a healthcare setting, it is possible for HAES® to improve health provider bias, and fat patient avoidance of medical professionals or health measures. In order for this to occur, health providers would need to set aside their pre-conceived assumptions or even prejudices about fat patients, and instead seek out the strong evidence pointing to the inefficacy of long-term weight loss in fat patients (Miller, 1999; Howard et al., 2006; Mann et al., 2007; Tylka et al., 2014), and stop prescribing weight loss as a solution to being fat, or as a solution to health problems.

If health professionals embrace HAES®, move the focus off weight, and onto health behaviors, and perhaps metabolic measures of health rather than weight loss, they give their patients a chance to focus on behaviors that can improve their health. If a fat person eats a balanced diet, engages in movement they enjoy, reduces stress, and engages in self-care activities but still remains fat, then prescribing weight loss implies they should then eat an unbalanced starvation diet, embark on exercise that isn't integrated or able to be maintained in their life, experience a sense of failure at being unable to control whether the scales register weight loss, and eventually avoid healthcare providers when the weight regain happens. The evidence that prescribing weight loss does not work has been available for many years now (Miller, 1999; Howard et al., 2006; Mann et al., 2007).

Health providers would then need to engage with the evidence that health-seeking behaviors are more effective than weight loss goals for those patients who are willing to seek health. Health-seeking behaviors are also possible for everyone, and within an individual's control, and don't rely on whether the numbers on the scale change from week-to-week. In other words, weight loss is not possible for everyone, and sustained and maintained weight loss are generally a failure for most people, but health-seeking behaviors are possible to implement, where the patient seeks to improve their health.

HAES® also has the potential to change the way disordered eating is treated in fat patients, by bringing the focus onto healthy behaviors rather than weight. While the focus is on weight, and the assumption is that it is always good for a fat person to lose weight, no matter their technique or behaviors, disordered eating, weight cycling, anxiety, and patients hiding their health behaviors for fear of being shamed, will continue. The increase of eating disorders in Western countries, especially amongst younger and younger girls, coincides with the increase in anti-obesity messages, anti-fat stigma, and focus on weight. For example, eating disorders leading to hospitalizations rose by 119 percent for children under 12 years of age in the period 1999-2006 (Zhao and Encinosa, 2009). There is now evidence that HAES® does work to decrease incidents of eating disorders and disordered eating, provide a more sustainable and realistic model for ongoing health goals, and improve the quality of life and well-being for fat people (Bacon et al., 2005; Carroll et al., 2007; Provencher et al., 2007; Gagnon-Girouard et al., 2010).

However, at times, HAES® is still often presented, especially in short-hand, in reaction to the current dominant health paradigm, hence only in terms of exercise and eating, and in terms of a notion that we are aiming, and should be aiming for “health,” without really addressing what “health” is and is not and who is allowed to be considered “healthy.”

In a neoliberal environment, assumptions are made that nutritious and affordable food is available for everyone, that people have the time to engage in enjoyable physical movement, and have the finances to support their food and exercise choices and time needs. All of these assumptions pose potential problems, and could exclude those fat individuals who are outside these privileged assumptions.

When the HAES® model is discussed in public forums, there is often still the assumption that good citizens want to engage in health-seeking behaviors, despite the principles directly stating that “ASDAH's HAES Principles reject judgments about health and any discourse of individual *responsibility* around health, in favor of a discourse of individualized health *needs*” (ASDAH, 2016). When applied by the health profession, privileged assumptions are made at times in the expectations of how people will seek health, both by the dominant health paradigm and the HAES® paradigm. The underlying assumption is that fat people are middle-class, white, financially stable, and living in urban locations, and therefore able to access “healthy” food and lifestyles. In Western cultural ideas about health-seeking behaviors, there is barely a mention of barriers to health for people who live with chronic disease, super-fat people with fat “aprons” that reach to their knees and have mobility problems, the mentally ill, homeless and abandoned in our culture, new mothers with post-natal depression, the segregation and institutionalization of our elderly populations, and the living conditions and shorter average life spans within indigenous communities in many countries, such as Canada. However, efforts have been made by the HAES® movement to modify their original mission to be more inclusive (HAES® Principles, 2013) and to acknowledge that accessing healthy behaviors and high quality health care is not possible for all people, even in the Western world.

As Stacey Nye writes, on the Association for Size Diversity & Health (*ASDAH*) HAES® blog: “Being healthy takes *time, effort, and money*, and it's no one's business but your own whether you choose to engage in healthy behaviors” [our emphasis](Nye, 2015). This individual time, effort and money is not often acknowledged by the dominant discourse about health.

While HAES® proponents deconstruct the notion that weight is within an individual's control, *health* at times is still presented as within an individual's control. Bacon states, “Turn over control to your body and you will settle at a healthy weight. And regardless of whether you do lose weight, your health and well-being will markedly improve. You will find that biology is much more powerful than willpower” (Bacon, 2010). Predictive talk about health and weight is something that medical professionals often do, and it is problematic because we do not control our health, no matter how “good” we are or how many health-seeking behaviors we engage in. It is why people say “that's so unfair” when someone who is able-bodied gets cancer or has a heart attack, because the striving for health is also a striving for control, and an aversion to death. It is a hope that by engaging in traditional health-seeking behavior, usually eating in a “disciplined” manner and exercising regularly, you can avoid sickness and early death.

For example, Jenny was advised to increase her physical activity and eat more low GI foods in order to improve her blood sugar numbers. This was her experience of the unexpected results:

*Diet and exercise have not had the desired effect of improving my blood sugar numbers. When I was diagnosed, I increased my level of exercise, joined a low-pressure gym with a fat-friendly manager (yes, a slight miracle, I know) and changed my eating habits—usually by eating more than I was, and eating more regularly, with slightly more focus on low-GI foods. But my blood sugar levels got worse despite this. So, after discussion with the dietician, we've identified that reducing stress and increasing my sleep are the targets. But, what if it's out of my control? This notion that eating well and exercising regularly can make you healthy whether you're fat or thin is wrong in some cases. I've had to accept all over again that I don't control my body and its internal functions (2013)*.

In this case, Jenny was relieved that the HAES® dietician that she researched and located believed her account of eating and exercising, rather than assuming Jenny was reporting inaccurately, as some medical professionals have accused her of in the past.

Even when it tries to be as inclusive as possible, one model can't necessarily encompass everyone's needs. We can ask questions such as where does HAES® fit within a non-Western context, in countries that are war-torn or rife with poverty? Are we asking too much of HAES®? Perhaps, but in a model (or movement) that purports to support all bodies, the limitations and exclusions need to be acknowledged and discussed.

While somewhat problematic, we suggest HAES® is very useful, and that HAES® principles can do the most good as a model of health for medical professionals to use in their practice. When in application, those working with HAES® needs to ensure: that it doesn't fall into a “healthism” paradigm, that it is inclusive and accessible, that it fully considers structural health barriers in society, that it acknowledges in meaningful ways the intersections of other identities and prejudices, and that a broader idea of what health is about is acknowledged properly.

## Conclusion

The label of healthy is reserved for those who actively pursue health through conspicuous consumption; the latest diet, the latest workout, the latest procedure. In turn, health has become a commodity. An understanding of health, and how to attain it, that primarily focuses on an individual striving to be healthy, is an individualistic model that some people are potentially excluded from. For example, the Australian Health Ministers' Advisory Council (2015) has identified the levels of fatness in the indigenous peoples of Australia as a key area of concern for population health. However, little attention seems to be paid to the barriers to health for indigenous peoples, fat or not. There are over 1000 indigenous communities in remote or very remote areas of Australia and only 175 community food stores that provide food for these communities (Pollard, 2012). Some of these communities don't have access to fresh vegetables at some times of the week, and many pay 53% more for their groceries (Department of Health Northern Territory Government, 2014). Many of these remote indigenous communities have a population of <100 people (Pollard, 2012) and therefore don't have sporting centers, or gyms with free childcare, or access to a psychologist or swimming and yoga classes. This lack of consideration of structural and societal influences on health needs to be further acknowledged and further documented in research. As Cheek (2008) notes, “We can now speak of being good or bad, or strong or weak in terms of our health behaviors, of making responsible or irresponsible choices” (981). In our experience, for fat people, it doesn't matter if you are bad with a “fatty” disease, or if you are in “good metabolic health” (but *NOT FOR LONG*, according to several medical professionals), the discrimination, humiliation, and stigma, from health care providers is the same. The fact that we, and every fat person we know, have experienced this fat stigma, no matter what their health status, is an indictment on the health care profession. Health care providers, public health policy makers, and institutions of health such as hospitals have substantial work to do if they exist to treat all patients, and improve the quality of life for all patients, rather than deterring and deferring appropriate health care and reducing quality of life through fat stigma, shame, and eventual patient avoidance of health care providers.

The topic of this special issue is “Obesity stigma in healthcare: impacts on policy, practice, and patients.” From a neoliberal perspective, policy comes first and this then impacts practice; the patients are simply recipients of the practice. We argue that we must center the lived experiences of the patients, to develop best practice, and devise ethical policy. We've purposefully shifted the focus from fat people's responsibility of health, to instead examine structural barriers and attitudes and practices that impact on fat people's health. In this way, we've rejected neoliberal assumptions of who is responsible for health when we speak about fatness. We used our lived experiences through autoethnography to illustrate how fat stigma impacts on the health of fat people. Sharing these stories, however, involves a level of risk. Risks included what to disclose to a large audience, such as Jenny's diabetes and Cat's immigration woes (experiences that haven't been shared with many close to us, including some family and our co-workers). Risks also included the shame and vulnerability that often accompanied relieving past experiences. As (Chatham-Carpenter, 2010) observes, “Autoethnographers have to be willing to do the hard work of feeling the pain and learning through the process of writing” (p. 9). Writing this together though, allowed for our vulnerability and pain to be shared with each other and with our writing; and in a way, lessened, through our acknowledged shared histories. Autoethnography can be especially useful for building scholarships of those on the margins; it allows us to be agentic producers of our own knowledge, and allows for “a reflexive attempt to construct meaning in our lives and heal or grow from our pain” (Ellis, 2007, p. 26).

Our experiences and understandings of fat health should not be taken as illustration of the experiences and understandings for all fat people. We are white cis-gendered, well-educated, corporate class, women living in Western countries. The experiences and understandings of other fat people, especially those located at intersections of oppressions (be they race, class, ability, sexual orientation, etc.), may indeed present very differently. Further research needs to be undertaken to elucidate the range of experiences and understandings that fat people in these groups may be living.

As we conclude our writing, we encourage those engaging with this special issue to reflect back on the many questions we have posed throughout this piece: Is HAES an appropriate alternative to weight-based models of health? Do we prescribe for fat people what we diagnose as eating disorders in non-fat people? How can anti-fat attitudes be reduced in healthcare providers? Why do we prioritize physical health over other kinds of health, especially for fat people? And lastly, can fat people “have health”?

## Author contributions

Both authors contributed to all aspects of research, writing, and drafting the paper. Both JL and CP wrote autoethnographic sections, researched aspects of the arguments presented (divided 50-50), and both drafted, redrafted and provided final approval of the manuscript submitted here.

### Conflict of interest statement

The authors declare that the research was conducted in the absence of any commercial or financial relationships that could be construed as a potential conflict of interest. The reviewer LLS and the handling Editor declared their shared affiliation, and the handling Editor states that the process nevertheless met the standards of a fair and objective review

## Footnotes

^1^There are many measures designed to measure fat stigma, including The Fat Stereotypes questionnaire, the Anti-Fat Attitudes scale, the Anti-Fat Attitudes Test, and the Causes of Obesity scale (Riggs et al., 2010). The Weight Self-Stigma questionnaire (Lillis et al., 2010) reports a Cronbach's a of.878 and correlates highly using Cohen's cut-offs with similar measures, such as the Attitudes Toward Obese People (ATOP) scale and the Beliefs About Obese Persons (BAOP) scale (For info about ATOP and BAOP scales, see Allison et al., 1991).

## References

[B1] Adams C. H. Smith N. J. Wilbur D. C. Grady K. E. (1993). The relationship of obesity to the frequency of pelvic examinations: do physician and patient attitudes make a difference? Women Health 20, 45–57. 8372479 10.1300/J013v20n02_04

[B2] Aldrich T. Hackley B. (2010). The impact of obesity on gynecologic cancer screening: an integrative literature review. J Midwifery Womens Health 55, 344–356. 10.1016/j.jmwh.2009.10.001 20630361

[B3] Allison D. B. Basile V. C. Yuker H. E. (1991). The measurement of attitudes toward and beliefs about obese persons. Int. J. Eat. Disord. 10, 599–607. 10.1002/1098-108X(199109)10:5<599::AID-EAT2260100512>3.0.CO;2

[B4] Ambwani S. Thomas K. M. Hopwood C. J. Moss S. A. Grilo C. M. (2014). Obesity stigmatization as the status quo: structural considerations and prevalence among young adults in the US. Eat. Behav. 15, 366–370. 10.1016/j.eatbeh.2014.04.005 25064282

[B5] Amonkar M. M. Madhavan S. (2002). Compliance rates and predictors of cancer screening recommendations among Appalachian women. J. Health Care Poor Underserved 13, 443–460. 10.1353/hpu.2010.0582 12407962

[B6] Amy N. K. Aalborg A. Lyons P. Keranen L. (2006). Barriers to routine gynecological cancer screening for White and African-American obese women. Int. J. Obes. 30, 147–155. 10.1038/sj.ijo.0803105 16231037

[B8] Association for size diversity and health (ASDAH) (2016). HAES® Principles. Available online at: https://www.sizediversityandhealth.org/content.asp?id=76

[B9] Australian Health Ministers Advisory Council (2015). Aboriginal and Torres Strait Islander Health Performance Framework 2014 Report. AHMAC, Canberra.

[B7] Bacon L. (2010). Health at Every Size: The Surprising Truth about Your Weight. Dallas, TX: BenBella Books.

[B10] Bacon L. Aphramor L. (2011). Weight science: evaluating the evidence for a paradigm shift. Nutr. J. 10:9. 10.1186/1475-2891-10-9 21261939 PMC3041737

[B11] Bacon L. Stern J. S. Van Loan M. D. Keim N. L. (2005). Size acceptance and intuitive eating improve health for obese, female chronic dieters. J. Am. Diet. Assoc. 105, 929–936. 10.1016/j.jada.2005.03.011 15942543

[B12] Barlösius E. Philipps A. (2015). Felt stigma and obesity: introducing the generalized other. Soc. Sci. Med. 130, 9–15. 10.1016/j.socscimed.2015.01.048 25658623

[B13] Berryman D. E. Dubale G. M. Manchester D. S. Mittelstaedt R. (2006). Dietetics students possess negative attitudes toward obesity similar to nondietetics students. J. Am. Diet. Assoc. 106, 1678–1682. 10.1016/j.jada.2006.07.016 17000203

[B14] Bordo S. (2009). Not just a “white girl's thing”: the changing face of food and body image problems, in Critical Feminist Approaches to EDs, eds Malson H. Burns M. (London: Routledge/Taylor & Francis Group), 46–59.

[B15] Braveman P. Gruskin S. (2003). Defining equity in health. J. Epidemiol. Community Health 57, 254–258. 10.1136/jech.57.4.254 12646539 PMC1732430

[B16] Brewis A. A. Wutich A. Falletta-Cowden A. Rodriguez-Soto I. (2011). Body norms and fat stigma in global perspective. Curr. Anthropol. 52, 269–276. 10.1086/659309

[B17] Brown I. McClimens A. (2012). Ambivalence and obesity stigma in decisions about weight management: a qualitative study. Sci. Res. 4, 1562–1569. 10.4236/health.2012.412a224

[B18] Brown I. Stride C. Psarou A. Brewins L. Thompson J. (2007). Management of obesity in primary care: nurses' practices, beliefs and attitudes. J. Adv. Nurs. 59, 329–341. 10.1111/j.1365-2648.2007.04297.x 17635298

[B19] Brown I. Thompson J. Tod A. Jones G. (2006). Primary care support for tackling obesity: a qualitative study of the perceptions of obese patients. Br. J. Gen. Pract. 56, 666–672. 16953998 PMC1876632

[B20] Bryne S. K. (2008). Healthcare avoidance: a critical review. Holist. Nurs. Pract. 22, 280–292. 10.1097/01.HNP.0000334921.31433.c6 18758277

[B21] Budd G. M. Mariotti M. Graff D. Falkenstein K. (2011). Health care professionals' attitudes about obesity: an integrative review. Appl. Nurs. Res. 24, 127–137. 10.1016/j.apnr.2009.05.001 20974067

[B22] Burgard D. (2004). Does one theoretical approach fit all? HAES and size diversity. Health Every Size J. 18, 43–46.

[B23] Burgard D. (2006). We should know better. Health Every Size J, 20, 83–88.

[B24] Burgard D. (2009). What is “health at every size”?, in The Fat Studies Reader, eds Rothblum E. Solovay S. (New York, NY: New York University Press), 41–53.

[B25] Burgard D. (2013). Weight Stigma Viewed through the Eating Disorders lens. Binge Eating Disorder Association website. Available online at: http://bedaonline.com/wsaw2013/weight-stigma-viewed-eating-disorders-lens-deb-burgard/

[B26] Burr V. (2015). Social Constructionism, 3rd edn London: Routledge.

[B27] Buxton B. K. Snethen J. (2013). Obese women's perceptions and experiences of healthcare and primary care providers: a phenomenological study. Nurs. Res. 62, 252–259. 10.1097/NNR.0b013e318299a6ba 23817283

[B28] Callahan D. (1973). The WHO defintion of health. Hastings Cent. Stud. 1, 77–87. 10.2307/3527467 4607284

[B29] Campbell K. Engel H. Timperio A. Cooper C. Crawford D. (2000). Obesity management: australian general practitioners' attitudes and practices. Obes. Res. 8, 459–466. 10.1038/oby.2000.57 11011913

[B30] Carney P. A. Harwood B. G. Weiss J. E. Eliassen M. S. Goodrich M. E. (2002). Factors associated with interval adherence to mammography screening in a population-based sample of New Hampshire women. Cancer 95, 219–227. 10.1002/cncr.10681 12124819

[B31] Carroll S. Borkoles E. Polman R. (2007). Short-term effects of a non-dieting lifestyle intervention program on weight management, fitness, metabolic risk, and psychological well-being in obese premenopausal females with the metabolic syndrome. Appl. Physiol. Nutr. Metab. 32, 125–142. 10.1139/h06-093 17332789

[B32] Chatham-Carpenter A. (2010). Do thyself no harm. J. Res. Pract. 6, 1.

[B33] Cheek J. (2008). Healthism: a new conservatism? Qual. Health Res. 19, 974–982. 10.1177/1049732308320444 18552323

[B34] Cohen S. S. Signorello L. B. Gammon M. D. Blot W. J. (2007). Obesity and recent mammography use among black and white women in the Southern Community Cohort Study (United States). Cancer Causes Control 18, 765–773. 10.1007/s10552-007-9019-3 17569015

[B35] Cooper C. (2016). Fat Activism: A Radical Social Movement. Bristol: HammerOn Press.

[B36] Council S. K. Placek C. (2014). Cultural change and explicit anti-fat attitudes in a developing nation: a case study in rural Dominica. Soc. Med. 9, 11–21.

[B37] Crawford R. (1980). Healthism and the medicalization of everyday life. Int. J. Health Serv. 10, 365–388. 10.2190/3H2H-3XJN-3KAY-G9NY 7419309

[B38] Creel E. Tillman K. (2011). Stigmatization of overweight patients by nurses. Qual. Rep. 16, 1330–1351.

[B39] Culbert K. M. Racine S. E. Klump K. L. (2015). Research review: what we have learned about the causes of eating disorders – a synthesis of sociocultural, psychological, and biological research. J. Child Psychol. Psychiatry 56, 1141–1164. 10.1111/jcpp.12441 26095891

[B40] Dedeli O. Bursalioglu S. A. Deveci A. (2014). Validity and reliability of the Turkish version of the attitudes toward obese persons scale and the beliefs about obese persons scale. Clin. Nurs. Stud. 2:p105 10.5430/cns.v2n4p105

[B41] DeJong W. (1980). The stigma of obesity: the consequence of naïve assumptions concerning the causes of physical deviance. J. Health Soc. Behav. 21, 75–87. 10.2307/2136696 7365232

[B42] Department of Health Northern Territory Government (2014). Northern Territory Market Basket Survey Report. Available online at: http://digitallibrary.health.nt.gov.au/prodjspui/bitstream/10137/616/2/Northern%20Territory%20%20Market%20Basket%20Survey%20report%202014.pdf

[B43] Destounis S. Newell M. Pinsky R. (2011). Breast imaging and intervention in the overweight and obese patient. Am. J. Roentgenol. 196, 296–302. 10.2214/AJR.10.5556 21257879

[B44] Donnelly L. (2016). Girls with Anorexia Turned Away by NHS Because They are ‘Not Thin Enough’. The Telegraph, U. K. Available online at: http://www.telegraph.co.uk/news/2016/08/03/girls-with-anorexia-turned-away-by-nhs-because-not-thin-enough/

[B45] Drury C. A. Aramburu C. Louis M. (2002). Exploring the association between body weight, stigma of obesity, and health care avoidance. J. Am. Acad. Nurse Pract. 14, 554–561. 10.1111/j.1745-7599.2002.tb00089.x 12567923

[B46] Ellis C. (2007). Telling secrets, revealing lives: Relational ethics in research with intimate others. Qual. Inq. 13, 3–29. 10.1177/1077800406294947

[B47] Ellis C. Adams T. E. Bochner A. P. (2011). Autoethnography: an overview. Hist. Soc. Res. 273–290.

[B48] Eriksen S. J. Manke B. (2011). “Because being fat means being sick”: children at risk of Type 2 diabetes. Sociol. Inq. 81, 549–569. 10.1111/j.1475-682X.2011.00392.x 22171368

[B49] Ferrante J. M. Fyffe D. C. Vega M. L. Piasecki A. K. Ohman-Strickland P. A. Crabtree B. F. (2010). Family physicians' barriers to cancer screening in extremely obese patients. Obesity 18, 1153–1159. 10.1038/oby.2009.481 20019676 PMC2953250

[B50] Ferrante J. M. Ohman-Strickland P. Hudson S. V. Hahn K. A. Scott J. G. Crabtree B. F. (2006). Colorectal cancer screening among obese versus non-obese patients in primary care practices. Cancer Detect. Prev. 30, 459–465. 10.1016/j.cdp.2006.09.003 17067753 PMC1761119

[B51] Forhan M. Salas X. R. (2013). Inequities in healthcare: a review of bias and discrimination in obesity treatment. Can. J. Diabetes 37, 205–209. 10.1016/j.jcjd.2013.03.362 24070845

[B52] Forhan M. Risdon C. Solomon P. (2013). Contributors to patient engagement in primary health care: perceptions of patients with obesity. Prim. Health Care Res. Dev. 14, 367–372. 10.1017/S1463423612000643 23237022

[B53] Foster G. D. Wadden T. A. Makris A. P. Davidson D. Sanderson R. S. Allison D. B.. (2003). Primary care physicians' attitudes about obesity and its treatment. Obes. Res. 11, 1168–1177. 10.1038/oby.2003.161 14569041

[B54] Frank A. (1993). Futility and avoidance: medical professionals in the treatment of obesity. JAMA 269, 2132–2133. 10.1001/jama.1993.03500160102041 8468770

[B55] Gagnon-Girouard M. P. Bégin C. Provencher V. Tremblay A. Mongeau L. Boivin S.. (2010). Psychological impact of a “Health-at-every-size” intervention on weight-preoccupied overweight/obese women. J. Obes. 1660–1665. 10.1155/2010/928097 20798861 PMC2925467

[B56] Geist-Martin P. (2010). Exemplifying collaborative autoethnographic practice via shared stories of mothering. J. Res. Pract. 6, 1–14.

[B57] Goffman E. (1963). Stigma: Notes on a Spoiled Identity. Englewood Cliffs, NJ: Prentice Hall.

[B58] Green S. M. McCoubrie M. Cullingham C. (2000). Practice nurses' and health visitors' knowledge of obesity assessment and management. J. Hum. Nutr. Diet. 13, 413–423. 10.1046/j.1365-277X.2000.00256.x

[B59] Greener J. Douglas F. van Teijlingen E. (2010). More of the same? Conflicting perspectives of obesity causation and intervention amongst overweight people, health professionals and policy makers. Soc. Sci. Med. 70, 1042–1049. 10.1016/j.socscimed.2009.11.017 20106576

[B60] HAES® Principles (2013). Association for Size Diversity and Health. Available online at: https://www.sizediversityandhealth.org/content.asp?id=152

[B61] Hatzenbuehler M. L. Phelan J. C. Link B. G. (2013). Stigma as a fundamental cause of population health inequalities. Am. J. Public Health 103, 813–821. 10.2105/AJPH.2012.301069 23488505 PMC3682466

[B62] Hebl M. R. Xu J. (2001). Weighing the care: physicians' reactions to the size of a patient. Int. J. Obes Relat. Metab Disord. 25, 1246–1252. 10.1038/sj.ijo.0801681 11477511

[B63] Hebl M. R. Xu J. Mason M. F. (2003). Weighing the care: patients' perceptions of physician care as a function of gender and weight. Int. J. Obes. 27, 269–275. 10.1038/sj.ijo.802231 12587009

[B64] Heuer C. A. McClure K. J. Puhl R. M. (2011). Obesity stigma in online news: a visual content analysis. J. Health Commun. 16, 976–987. 10.1080/10810730.2011.561915 21541876

[B65] Hilbert A. Braehler E. Haeuser W. Zenger M. (2014). Weight bias internalization, core self-evaluation, and health in overweight and obese persons. Obesity 22, 79–85. 10.1002/oby.20561 23836723

[B66] Himes S. M. Thompson J. K. (2007). Fat stigmatization in television shows and movies: a content analysis. Obesity 15, 712–718. 10.1038/oby.2007.635 17372322

[B67] Howard B. V. Manson J. E. Stefanick M. L. Beresford S. A. Frank G. Jones B. Prentice R.. (2006). Low-fat dietary pattern and weight change over 7 years: the women's health initiative dietary modification trial. J. Am. Med. Assoc. 295, 39–49. 10.1001/jama.295.1.39 16391215

[B68] Huber M. Knottnerus J. A. Green L. van der Horst H. Jadad A. R. Kromhout D.. (2011). How should we define health? Br. Med. J. 343, 1–3. 10.1136/bmj.d4163 21791490

[B69] Huizinga M. M. Cooper L. A. Bleich S. N. Clark J. M. Beach M. C. (2009). Physician respect for patients with obesity. J. Gen. Intern. Med. 24, 1236–1239. 10.1007/s11606-009-1104-8 19763700 PMC2771236

[B70] Hunger J. M. Major B. Blodorn A. Miller C. T. (2015). Weighed down by stigma: how weight-based social identity threat contributes to weight gain and poor health. Soc. Pers. Psychol. Compass 9, 255–268. 10.1111/spc3.12172 PMC5720363 29225670

[B71] Hunt K. A. Sickles E. A. (2000). Effect of obesity on screening mammography: outcomes analysis of 88,346 consecutive examinations. Am. J. Roentgenol. 174, 1251–1255. 10.2214/ajr.174.5.1741251 10789771

[B72] Ingraham N. Roberts S. C. Weitz T. A. (2014). Prior family planning experiences of obese women seeking abortion care. Womens Health Issues 24, e125–e130. 10.1016/j.whi.2013.10.008 24439938

[B73] Jeffrey C. A. Kitto S. (2006). Struggling to care: nurses' perceptions of caring for obese patients in an Australian bariatric ward. Health Sociol. Rev. 15, 71–83. 10.5172/hesr.2006.15.1.71

[B74] Johnston C. A. (2012). The impact of weight-based discrimination in the health care setting. Am. J. Lifestyle Med. 6, 452–454. 10.1177/1559827612456348

[B75] Jutel A. G. (2005). Weighing health: the moral burden of obesity. Soc. Semiotics 15, 113–125. 10.1080/10350330500154717

[B76] Jutel A. G. (2014). Putting a Name to It: Diagnosis in Contemporary Society. Baltimore, MD: JHU Press.

[B77] Kreuter M. W. Scharff D. P. Brennan L. K. Lukwago S. N. (1997). Physician recommendations for diet and physical activity: which patients get advised to change? Prev. Med. 26, 825–833. 9388794 10.1006/pmed.1997.0216

[B78] Latner J. D. Stunkard A. J. (2003). Getting worse: the stigmatization of obese children. Obes. Res. 11, 452–456. 10.1038/oby.2003.61 12634444

[B79] Lavoie K. L. Bacon S. L. Labrecque M. Cartier A. Ditto B. (2006). Higher BMI is associated with worse asthma control and quality of life but not asthma severity. Respir. Med. 100, 648–657. 10.1016/j.rmed.2005.08.001 16159709

[B80] LeBesco K. (2009). Weight management, good health and the will to normality, in Critical Feminist Approaches to Eating Dis/orders, eds Malson H. Burns M. (New York, NY: Routledge), 146–156.

[B81] Lee J. (2012). Not just a type: diabetes, fat and fear. Somatechnics 2, 80–83. 10.3366/soma.2012.0041

[B82] Lee J. (2015). All the way from b(lame) to a(cceptance): diabetes, fat and fat activism, in The Politics of Size: Perspectives from the Fat Acceptance Movement, Vol. 2, ed Chastain R. (Santa Barbara, CA: Praeger), 63–73.

[B83] Lewis S. Thomas S. L. Blood R. W. Castle D. J. Hyde J. Komesaroff P. A. (2011). How do obese individuals perceive and respond to the different types of obesity stigma that they encounter in their daily lives? A qualitative study. Soc. Sci. Med. 73, 1349–1356. 10.1016/j.socscimed.2011.08.021 21944718

[B84] Lillis J. Luoma J. B. Levin M. E. Hayes S. C. (2010). Measuring weight self-stigma: the weight self-stigma questionnaire. Obesity 18, 971–976. 10.1038/oby.2009.353 19834462

[B85] Malterud K. Ulriksen K. (2010). Obesity in general practice: a focus group study on patient experiences. Scand. J. Prim. Health Care 28, 205–210. 10.3109/02813432.2010.526773 20942741 PMC3444791

[B86] Mann T. Tomiyama A. J. Westling E. Lew A. M. Samuels B. Chatman J. (2007). Medicare's search for effective obesity treatments: diets are not the answer. Am. Psychol. Assoc. 62, 220–233. 10.1037/0003-066X.62.3.220 17469900

[B87] Maroney D. Golub S. (1992). Nurses' attitudes toward obese persons and certain ethnic groups. Percept. Motor Skills 75, 387–391. 1408594 10.1177/003151259207500201

[B88] McCabe M. P. Ricciardelli L. A. (2005). A prospective study of pressures from parents, peers, and the media on extreme weight change behaviors among adolescent boys and girls. Behav. Res. Ther. 43, 653–668. 10.1016/j.brat.2004.05.004 15865919

[B89] McCarthy A. (2014,. 28 February). I'm a Fat Woman Who Had an Eating Disorder, and No, the Two are Not Mutually Exclusive. Bustle. Available online at: http://www.bustle.com/articles/16685-im-a-fat-woman-who-had-an-eating-disorder-and-no-the-two-are-not-mutually.,

[B90] McCubbin J. (2016). Eating Disorders: Patients with “Wrong Weight” Refused Care. BBC News, U. K. Available online at: http://www.bbc.com/news/health-36956849

[B91] Mercer S. W. Tessier S. (2001). A qualitative study of general practitioners' and practice nurses' attitudes to obesity management in primary care. Health Bull. 59, 248–253. 12664735

[B92] Merrill E. Grassley J. (2008). Women's stories of their experiences as overweight patients. J. Adv. Nurs. 64, 139–146. 10.1111/j.1365-2648.2008.04794.x 18764854

[B93] Metzl J. M. Kirkland A. (2010). Against Health: How Health Became the New Morality. New York, NY: New York University Press.

[B94] Miller W. C. (1999). How effective are traditional dietary and exercise interventions for weight loss? Med. Sci. Sports Exerc. 31, 1129–1134. 10449014 10.1097/00005768-199908000-00008

[B95] Mitchell R. S. Padwal R. S. Chuck A. W. Klarenbach S. W. (2008). Cancer screening among the overweight and obese in Canada. Am. J. Prev. Med. 35, 127–132. 10.1016/j.amepre.2008.03.031 18617081

[B96] Montani J. P. Viecelli A. K. Prévot A. Dulloo A. G. (2006). Weight cycling during growth and beyond as a risk factor for later cardiovascular diseases: the “repeated overshoot” theory. Int. J. Obes. 30, 58–66. 10.1038/sj.ijo.0803520 17133237

[B97] Muenning P. (2008). The relationship between stigma and obesity-associated disease. BMC Public Health 8, 1–10. 10.1186/1471-2458-8-128 18426601 PMC2386473

[B98] New Zealand Immigration (2015). Health Requirements for Entry to New Zealand. Ministry of Business, Innovation & Employment Wellington, New Zealand.

[B99] Ngunjiri F. W. Hernandez K. A. C. Chang H. (2010). Living autoethnography: connecting life and research. J. Res. Pract. 6, 1.

[B100] Nye S. (2015). The HAES® Files: I am No Shadow. Health at Every Size® Blog, the Association for Size Diversity and Health. Available online at: https://healthateverysizeblog.org/tag/beauty/

[B101] Ogden J. Clementi C. (2010). The experience of being obese and the many consequences of stigma. J. Obes. 2010:429098. 10.1155/2010/429098 20721360 PMC2915811

[B102] Olson C. L. Schumaker H. D. Yawn B. P. (1994). Overweight women delay medical care. Arch. Fam. Med. 3:888. 10.1001/archfami.3.10.888 8000560

[B103] Olson M. B. Kelsey S. F. Bittner V. Reis S. E. Reichek N. Handberg E. M.. (2000). Weight cycling and high-density lipoprotein cholesterol in women: Evidence of an adverse effect: a report from the NHLBI-sponsored WISE study. Women's Ischemia Syndrome Evaluation Study Group. J. Am. Coll. Cardiol. 36, 1565–1571. 10.1016/S0735-1097(00)00901-3 11079659

[B104] Østbye T. Taylor D. H. Jr. Yancy W. S. Jr. Krause K. M. (2005). Associations between obesity and receipt of screening mammography, Papanicolaou tests, and influenza vaccination: results from the Health and Retirement Study (HRS) and the Asset and Health Dynamics Among the Oldest Old (AHEAD) Study. Am. J. Public Health 95, 1623–1630. 10.2105/AJPH.2004.047803 16051935 PMC1449407

[B105] Pausé C. J. (2012). Live to tell: coming out as fat. Somatechnics 2, 42–56. 10.3366/soma.2012.0038

[B106] Pausé C. J. (2014). Die another day: the obstacles facing fat people in accessing quality healthcare. Narrative Inq. Bioeth. 4, 135–141. 10.1353/nib.2014.0039 25130353

[B107] Pausé C. J. (2015). Rebel heart: performing fatness wrong online. M/C J. 18.

[B108] Persky S. Eccleston C. P. (2011). Medical student bias and care recommendations for an obese versus non-obese virtual patient. Int. J. Obes. 35, 728–735. 10.1038/ijo.2010.173 20820169 PMC3000449

[B109] Phelan S. M. Burgess D. J. Yeazel M. W. Hellerstedt W. L. Griffin J. M. van Ryn M. (2015). Impact of weight bias and stigma on quality of care and outcomes for patients with obesity. Obes. Rev. 16, 319–326. 10.1111/obr.12266 25752756 PMC4381543

[B110] Phelan S. M. Dovidio J. F. Puhl R. M. Burgess D. J. Nelson D. B. Yeazel M. W. Ryn M. (2014). Implicit and explicit weight bias in a national sample of 4,732 medical students: the medical student CHANGES study. Obesity 22, 1201–1208. 10.1002/oby.20687 24375989 PMC3968216

[B111] Polivy J. Herman C. P. (2002). Causes of eating disorders. Annu. Rev. Psychol. 53, 187–213. 10.1146/annurev.psych.53.100901.135103 11752484

[B112] Pollard C. (2012). Selecting interventions for food security, in Food Security in Australia: Challenges and Prospects for the Future, eds Farmar-Bowers Q. Higgins V. Millar J. (New York, NY: Springer), 97–112.

[B113] Poon M. Y. Tarrant M. (2009). Obesity: attitudes of undergraduate student nurses and registered nurses. J. Clin. Nurs. 18, 2355–2365. 10.1111/j.1365-2702.2008.02709.x 19374692

[B114] Presnell K. Bearman S. K. Stice E. (2004). Risk factors for body dissatisfaction in adolescent boys and girls: a prospective study. Int. J. Eat. Disord. 36, 389–401. 10.1002/eat.20045 15558645

[B115] Provencher V. Bégin C. Tremblay A. Mongeau L. Boivin S. Lemieux S. (2007). Short-term effects of a “Health-At-Every-Size” approach on eating behaviors and appetite ratings. Obesity 15, 957–966. 10.1038/oby.2007.638 17426331

[B116] Puhl R. Brownell K. D. (2001). Bias, discrimination, and obesity. Obes. Res. 9, 788–805. 10.1038/oby.2001.108 11743063

[B117] Puhl R. M. Brownell K. D. (2006). Confronting and coping with weight stigma: an investigation of overweight and obese adults. Obesity 14, 1802–1815. 10.1038/oby.2006.208 17062811

[B118] Puhl R. M. Heuer C. A. (2010). Obesity stigma: important considerations for public health. Am. J. Public Health 100, 1019–1028. 10.2105/AJPH.2009.159491 20075322 PMC2866597

[B119] Puhl R. M. Latner J. D. (2007). Stigma, obesity, and the health of the nation's children. Psychol. Bull. 133:557. 10.1037/0033-2909.133.4.557 17592956

[B120] Rice C. (2009). How big girls become fat girls: the cultural production of problem eating and physical inactivity, in Critical Feminist Approaches to EDs, eds Malson H. Burns M. (New York, NY: Routledge), 97–109.

[B121] Riggs N. Chou C. P. Spruijt Metz D. Pentz M. A. (2010). Executive cognitive function as a correlate and predictor of child food intake and physical activity. Child Neuropsychol. 16, 279–292. 10.1080/09297041003601488 20234954

[B122] Rogge M. M. Greenwald M. Golden A. (2004). Obesity, stigma, and civilized oppression. Adv. Nurs. Sci. 27, 301–315. 10.1097/00012272-200410000-00006 15602281

[B123] Sabin J. A. Marini M. Nosek B. A. (2012). Implicit and explicit anti-fat bias among a large sample of medical doctors by BMI, race/ethnicity and gender. PLoS ONE 7:e48448. 10.1371/journal.pone.0048448 23144885 PMC3492331

[B124] Sadati A. K. Rahnavard F. Ebrahimzadeh N. Rezaei A. (2016). Obesity, lived experience, and the self: a qualitative study of overweight people in Southern Iran. Womens Health Bull. 3:e31127 10.17795/whb-31127

[B125] Sartorius N. (2006). Lessons from a 10-year global programme against stigma and discrimination because of an illness 1. Psychol. Health Med. 11, 383–388. 10.1080/13548500600595418 17130075

[B126] Schwartz M. B. Chambliss H. O. Brownell K. D. Blair S. N. Billington C. (2003). Weight bias among health professionals specializing in obesity. Obes. Res. 11, 1033–1039. 10.1038/oby.2003.142 12972672

[B127] Setchell J. Watson B. Jones L. Gard M. Briffa K. (2014). Physiotherapists demonstrate weight stigma: a cross-sectional survey of Australian physiotherapists. J. Physiother. 60, 157–162. 10.1016/j.jphys.2014.06.020 25084637

[B128] Setchell J. Watson B. Jones L. Gard M. (2015). Weight stigma in physiotherapy practice: patient perceptions of interactions with physiotherapists. Man. Ther. 20, 835–841. 10.1016/j.math.2015.04.001 25920342

[B129] Simoes E. J. Newschaffer C. J. Hagdrup N. Ali-Abarghoui F. Tao X. Mack N.. (1999). Predictors of compliance with recommended cervical cancer screening schedule: a population-based study. J. Community Health 24, 115–130. 10.1023/A:1018754307718 10202691

[B130] Stafford B. M. La Puma J. Schiedermayer D. L. (1989). One face of beauty, one picture of health: the hidden aesthetic of medical practice. J. Med. Philos. 14, 213–230. 10.1093/jmp/14.2.213 2671230

[B131] Starfield B. (2001). New paradigms for quality in primary care. Br. J. Gen. Pract. 51, 303. 11458485 PMC1313982

[B132] Stein J. Luppa M. Ruzanska U. Sikorski C. König H. H. Riedel-Heller S. G. (2014). Measuring negative attitudes towards overweight and obesity in the german population–psychometric properties and reference values for the german short version of the fat phobia scale (FPS). PLoS ONE 9:e114641. 10.1371/journal.pone.0114641 25474195 PMC4256451

[B133] Stephenson W. B. (2011). The Experiences of Obese African American Women and Their Utilization of Preventive Healthcare Services. Unpublished dissertation, Georgia State University.

[B134] Stone O. Werner P. (2012). Israeli dietitians' professional stigma attached to obese patients. Qual. Health Res. 22, 768–776. 10.1177/1049732311431942 22218267

[B135] Strohacker K. McFarlin B. K. (2010). Influence of obesity, physical inactivity, and weight cycling on chronic inflammation. Front. Biosci. 2, 98–104. 20036858 10.2741/e70

[B136] Stuber J. Meyer I. Link B. (2008). Stigma, prejudice, discrimination and health. Soc. Sci. Med. 67, 351. 10.1016/j.socscimed.2008.03.023 18440687 PMC4006697

[B137] Tait A. (2015). When You're Both Overweight and Anorexic. Broadly [online]. Available online at: https://broadly.vice.com/en_us/article/when-youre-both-overweight-and-anorexic

[B138] Thompson R. L. Thomas D. E. (2000). A cross-sectional survey of the opinions on weight loss treatments of adult obese patients attending a dietetic clinic. Int. J. Obes. Relat. Metab. Disord. 24, 164–170. 10.1038/sj.ijo.0801102 10702766

[B139] Troumbley R. (2013). Colonization.com: Empire building for a new digital age. East West Aff. 93–107.

[B140] Tylka T. L. Annunziato R. A. Burgard D. Daníelsdóttir S. Shuman E. Davis C. Calogero R. M. (2014). The weight-inclusive versus weight-normative approach to health: evaluating the evidence for prioritizing well-being over weight loss. J. Obes. 2014:983495. 10.1155/2014/983495 25147734 PMC4132299

[B141] Watkins P. Hugmever A. D. (2012). Teaching about eating disorders from a Fat Studies perspective. Transformations 23, 171–182.

[B142] Wee C. C. McCarthy E. P. Davis R. B. Phillips R. S. (2004). Obesity and breast cancer screening. J. Gen. Intern. Med. 19, 324–331. 10.1111/j.1525-1497.2004.30354.x 15061741 PMC1492197

[B143] Wee C. C. McCarthy E. P. Davis R. B. Phillips R. S. (2000). Screening for cervical and breast cancer: is obesity an unrecognized barrier to preventive care? Ann. Intern. Med. 132, 697–704. 10.1111/j.1526-0976.2001.51012-8.pp.x 10787362

[B144] Whitehead M. (1992). The concepts and principles of equity and health. Int. J. Health Serv. 22, 429–445. 10.2190/986L-LHQ6-2VTE-YRRN 1644507

[B145] Whitelocks S. (2013). “I Weighed 200lbs and was Anorexic”: How Obese Teenagers with Eating Disorders Go Unnoticed Because They're Not “Skin And Bones”. Daily Mail Australia [online]. Available online at: http://www.dailymail.co.uk/femail/article-2429971/Obese-teenagers-eating-disorders-unnoticed-size.html

[B146] World Health Organisation (WHO) (1948). WHO Definition of Health. Preamble to the Constitution of the World Health Organisation. Available online at: http://www.who.int/about/definition/en/print.html (Accessed 27 January 2016).

[B147] World Health Organisation (WHO) (1986). The Ottawa Charter for Health Promotion. Available online at: http://www.who.int/healthpromotion/conferences/previous/ottawa/en/ (Accessed 20 January 2016).

[B148] Wigton R. S. McGaghie W. C. (2001). The effect of obesity on medical students' approach to patients with abdominal pain. J. Gen. Intern. Med. 16, 262–265. 10.1046/j.1525-1497.2001.016004262.x 11318928 PMC1495198

[B149] Wills W. Backett-Milburn K. Gregory S. Lawton J. (2006). Young teenagers' perceptions of their own and others' bodies: a qualitative study of obese, overweight and ‘normal’ weight young people in Scotland. Soc. Sci. Med. 62, 396–406. 10.1016/j.socscimed.2005.06.014 16039024

[B150] Zhao Y. M. S. Encinosa W. (2009). Hospitalizations for Eating Disorders from 1999 to 2006. Agency for Healthcare Research and Quality (AHRQ). Available online at: http://www.hcup-us.ahrq.gov/reports/statbriefs/sb70.jsp 21510032

[B151] Zhu K. Wu H. Jatoi I. Potter J. Shriver C. (2006). Body mass index and use of mammography screening in the United States. Prev. Med. 42, 381–385. 10.1016/j.ypmed.2006.01.020 16516284

[B152] Zuzelo P. R. Seminara P. (2006). Influence of registered nurses' attitudes toward bariatric patients on educational programming effectiveness. J. Contin Educ. Nurs. 37, 65–73. 10.3928/00220124-20060201-02 16883670

